# Effectiveness and safety of TNF inhibitors in adults with juvenile idiopathic arthritis

**DOI:** 10.1136/rmdopen-2016-000273

**Published:** 2016-10-07

**Authors:** Lianne Kearsley-Fleet, Flora McErlane, Helen E Foster, Mark Lunt, Kath D Watson, Deborah P M Symmons, Kimme L Hyrich

**Affiliations:** 1 Arthritis Research UK Centre for Epidemiology, Manchester Academic Health Science Centre, The University of Manchester, Manchester, UK; 2 Paediatric Rheumatology, Great North Children's Hospital, Newcastle upon Tyne, UK; 3 Musculoskeletal Research Group, Institute Cellular Medicine, Newcastle University, Newcastle upon Tyne, UK; 4 NIHR Manchester Musculoskeletal Biomedical Research Unit, Central Manchester University Hospitals NHS Foundation Trust and University of Manchester Partnership, Manchester, UK

**Keywords:** Juvenile Idiopathic Arthritis, DMARDs (biologic), Rheumatoid Arthritis, Outcomes research

## Abstract

**Introduction:**

Many children with juvenile idiopathic arthritis (JIA) continue to have active disease into adulthood. Adults with JIA are a heterogeneous group, and the effects of tumour necrosis factor inhibitor (TNFi) therapies are not well described. This analysis aims to describe treatment outcomes among patients with JIA starting TNFi for the first time in adulthood.

**Methods:**

Patients with arthritis onset <16 years starting their first TNFi therapy were identified from the British Society of Rheumatology Biologics Register. Disease activity outcomes (using 28-joint Disease Activity Score (DAS28) and Health Assessment Questionnaire (HAQ)) are presented at 1 year after start of therapy according to disease pattern. Incidence rates (IR) of adverse events per 1000 person-years (pyrs) were calculated. Outcomes in patients with polyarticular JIA were compared with a cohort (weighted for age and gender) of patients with rheumatoid arthritis (RA).

**Results:**

In 443 adults with JIA starting a first TNFi, disease activity over 1 year improved across all measures. There were 58 first serious infections (IR 22.3/1000 pyrs); 4 cardiovascular events (IR 1.4/1000 pyrs); 11 uveitis events (IR 4.0/1000 pyrs) and 16 malignancies (IR 3.9/1000 pyrs). Compared with the weighted RA cohort, disease activity improvement was similar; malignancy rates were lower and uveitis rates much higher. While crude IR were similar, JIA patients had a lower risk of serious infection (HR 0.5 (95% CI 0.3 to 0.9)).

**Conclusions:**

This is the largest study to describe disease activity and safety outcomes in adults with JIA receiving TNFi. Disease activity improved after 1 year in all disease patterns, suggesting TNFi is an effective therapy in this population.

**Key messages**

**What is already known about this subject?**

- Tumour necrosis factor inhibitors (TNFi) have been shown to be an effective treatment for children with juvenile idiopathic arthritis (JIA) when started in childhood.
- Little is known about their risks and benefits when used for JIA for the first time in adulthood.

**What does this study add?**

- This study shows that TNFi are an effective therapeutic option for adults with JIA, with a safety profile similar to that seen in rheumatoid arthritis.

**How might this impact on clinical practice?**

- For adults with persistent or recurrent symptoms of JIA in adulthood, introducing a TNFi at this point can result in improvements across a range of disease manifestations.

## Background

Juvenile idiopathic arthritis (JIA) is the most common chronic inflammatory musculoskeletal disease of childhood,^1^ and the management of JIA in children has evolved markedly over the past decade, to include a more aggressive approach with the goal of early remission.^2^ ^3^ In the UK, the current evidence base (including clinical trials and observational data) has enabled the development of treatment guidelines for children with JIA.^4^ In particular, first-line treatment with methotrexate, including a trial of subcutaneous methotrexate, is recommended for children with more severe forms of arthritis with the addition of tumour necrosis factor inhibitors (TNFi) for non-responders or those who are intolerant.

JIA is not confined to childhood, and research (albeit much from the prebiological era) suggests at least a third of patients will continue to have active disease in adulthood.^5–10^ Increasingly, children are entering adulthood already receiving TNFi started in childhood, and the limited evidence base supports the ongoing benefits of this therapy.^11^ ^12^ In addition, however, there is a cohort of patients with JIA who may require a biologic for the first time in adulthood. For some, this will represent persistently active disease, with a proportion having been diagnosed in later adolescence. Others may have become lost to follow-up in late adolescence or during transition, representing later to adult services. A third group may represent patients who may have initially achieved remission during childhood and then flare again in adulthood. Finally, for many older adults with JIA, TNFi was not an option in childhood, having only been licensed in Europe in 2000.^13^ As opposed to the effects of TNFi in adults with rheumatoid arthritis (RA), for which effectiveness and safety outcomes are well described,^14^ the medical management of adults with JIA is not well defined, and the evidence base to support decision-making is scarce. Factors such as very long disease duration and an increasing number of comorbidities may result in different response patterns and safety profiles compared with both those seen in younger children as well as those seen in RA.^15–18^

To date, only one analysis has examined treatment response and short-term drug survival in 225 adults with JIA (155 with known International League Against Rheumatism^19^ (ILAR) subtype). This study,^20^ using data from the British Society for Rheumatology Biologics Register in Rheumatoid Arthritis (BSRBR-RA) found that a majority of patients did experience improvement in disease activity and physical function after the start of TNFi therapy. Although this preliminary report was encouraging, it was undertaken on a very small selection of patients restrained by an attempt to classify patients strictly according to ILAR subtype.

This analysis aims to (1) analyse outcomes, including changes in disease activity and short-term to medium-term safety outcomes in adults with JIA starting TNFi therapy for the first time, (2) compare outcomes across JIA disease patterns and (3) compare outcomes with those observed in adults starting TNFi therapy for RA.

## Methods

This analysis included patients from BSRBR-RA, a national cohort study established in 2001, to investigate the long-term safety of biologics in adults with rheumatic conditions.^21^ Although primarily a study of RA, patients with diagnoses of ankylosing spondylitis (AS), psoriatic arthritis (PsA) and other rheumatic diseases, including JIA, have also been recruited. Ethical approval for the BSRBR-RA was granted by the North West Multi-Centre Research Ethics Committee in December 2000. All patients provided written informed consent.

At the start of TNFi therapy, patient demographics and disease activity measures are collected by a rheumatology nurse including age, gender, diagnosis, year of disease onset, past and current antirheumatic therapies, the 28-joint Disease Activity Score (DAS28) and its individual components,^22^ Medical Outcomes Study 36-item Short Form Health Survey (SF-36),^23^ and Health Assessment Questionnaire (HAQ). JIA-specific measures such as active and limited joint count are not recorded. Comorbidities are identified from a tick-list (see table 1 for details). Follow-up data are collected from the medical record, 6-monthly for 3 years, then annually thereafter and include disease activity, changes to antirheumatic therapy including reasons and occurrence of adverse events. HAQ and SF-36 were collected directly from all patients, 6-monthly for 3 years, according to the original BSRBR protocol. All patients are flagged with the Health and Social Care Information Centre (HSCIC) for occurrence of death and malignancy, which were reported directly to the BSRBR-RA.

**Table 1:** Characteristics of 443 adult patients with JIA at point of starting their first TNFi

	All JIA patients Median (IQR) or Number (%)
Gender, female	331 (75%)
Age at treatment start, years	33 (24–41)
Age at disease onset, years	13 (9–15)
Disease duration, years	22 (13–31)
Ethnicity; white	338 (95%) N=356
On methotrexate	257 (58%)
On steroids	173 (39%)
Disease pattern	
Systemic onset	12 (3%)
Oligoarticular pattern	7 (2%)
Polyarticular pattern	327 (73%)*
Enthesitis-related arthritis	53 (12%)
Psoriatic arthritis	44 (10%)
TNFi at registration	
Etanercept	199 (45%)
Infliximab	137 (31%)
Adalimumab	101 (23%)
Certolizumab	6 (1%)
Disease activity	
28 tender joint count	14 (8–20) N=401
28 swollen joint count	10 (5–15) N=402
Patient global assessment (100 mm VAS)	75 (65–85) N=405
ESR, mm/hour	35 (19–56) N=402
CRP, mg/L	30 (10–52) N=210
DAS28	6.2 (5.5–7.0) N=405
HAQ (0–3)	1.9 (1.5–2.4) N=403
Total comorbidities†	N=423
None	227 (54%)
1	131 (31%)
≥2	65 (15%)
Smoking status	N=438
Current smoker	101 (23%)
Previous smoker	79 (18%)
Never smoked	258 (59%)

*150 patients with polyarticular pattern were rheumatoid factor positive (46% of polyarticular pattern, 33% of cohort overall).

†Comorbidities include; hypertension, angina, myocardial infarction, stroke, epilepsy, asthma, chronic bronchitis/emphysema, peptic ulcer, liver disease, renal disease, tuberculosis, demyelination, diabetes, hyperthyroidism, depression, cancer.

Presenting number (%), or median (IQR), including number with available data (N) where applicable.

CRP, C reactive protein; DAS28, 28-joint Disease Activity Score; ESR, erythrocyte sedimentation rate; HAQ, Health Assessment Questionnaire; JIA, juvenile idiopathic arthritis; TNFi, tumour necrosis factor inhibitor; VAS, visual analog scale.

Previous work in this cohort has identified that most patients with childhood onset arthritis were not classified as having JIA, but rather given an alternative ‘adult’ diagnosis (eg, RA, AS, PsA).^20^ Therefore, based on year of birth and year of symptom onset, patients with an estimated disease onset age of <16 years were identified from the cohort. Patients with a non-arthritis diagnosis were excluded (eg, vasculitis) and the others grouped according to disease pattern, using information available in their study file: oligoarticular course (BSRBR-RA diagnosis of pauciarticular or oligoarticular JIA, or <5 swollen or tender joints ever recorded in the database), PsA (BSRBR-RA diagnosis of PsA or systemic psoriasis ever recorded), enthesitis-related arthritis (ERA) (BSRBR-RA diagnosis of AS or ERA), systemic arthritis (BRSBR-RA diagnosis of systemic JIA or Still's disease) and polyarticular course (BSRBR-RA diagnosis of polyarticular JIA (extended, rheumatoid factor negative or positive polyarthrits), or ≥5 swollen or tender joints ever recorded in the database). For adults with systemic arthritis or oligoarticular-course JIA, further information was obtained from their rheumatologist confirming their disease pattern, as the BSRBR-RA does not record systemic features and only records a 28-joint count.

The primary analysis included all patients with JIA starting their first TNFi therapy within 6 months of registration with the BSRBR-RA. Disease activity data at 1 year were analysed and outcomes included the absolute change from baseline (treatment start) in DAS28, HAQ^24^ and SF-36; the proportion of patients achieving the European League Against Rheumatism (EULAR) response^25^ and EULAR remission (DAS28<2.6) and the proportion of patients achieving a minimally clinical important difference (MCID) in HAQ of >0.22 units^26^ are presented. Kaplan-Meier curves were generated for survival on first TNFi therapy.

Rates of key serious adverse events (SAE) (infections, cardiovascular, uveitis, malignancies and deaths), with serious defined to include death, life threatening, hopsitalisation or disability, were determined. Only malignancies reported via the HSCIC or confirmed to the BSRBR-RA with histology were included. For deaths and malignancies, the exposure window began with the first dose of TNFi and continued until the event or 25 June 2014 (date of data lock for this analysis), regardless of changes to therapies (ie, an ever-exposed model). For other SAEs, the exposure window began with the first dose of TNFi and continued until the event, last returned follow-up form, or date of first missed dose of TNFi plus 90 days, whichever came first (ie, on-drug analysis). Crude incidence rates per 1000 person-years (pyrs) were calculated. Standardised incidence ratios (SIR) for malignancies and standardised mortality ratio (SMR) were calculated using UK population age and gender cancer and mortality rates provided by the Office for National Statistics (ONS).^27^

A cohort of 7877 patients in the BSRBR-RA with a physician diagnosis of RA and symptom onset ≥17 years old with the same age distribution as the JIA cohort at point of starting their first TNFi therapy was also identified and their outcomes were compared, using the same methods as the primary analysis, with adults with polyarticular JIA using an age-weighted and gender-weighted analysis. The RA patients were given a weight based on the ratio of JIA to RA patients for that age and gender group. For example, there were 14 female JIA patients aged 21 years old at registration, compared with 11 RA patients. Therefore, each of those 11 RA patients was given the weight of 14/11 (a weight >1). For females aged 48 years at registration, there were 3 JIA patients compared with 204 RA patients. Therefore, each of those 204 RA patients was given the weight of 3/204 (a weight <1). All JIA patients were given the weight of 1. As there were no male RA patients aged <20 years old and no female RA patients <18, males with JIA <20 years (n=6) and females with JIA <18 years (n=11) were weighted against RA patients aged 18 and 20, respectively. Baseline and effectiveness data were compared using linear and logistic regressions assuming analytic weights. Safety data were compared using Cox proportional hazards model assuming importance weights.

The analyses included all data available up to 30 November 2014 and were performed using Stata V.13 (StataCorp, College Station, Texas, USA).

## Results

In total, 553 patients with JIA were identified, of whom 443 were starting their first TNFi therapy; 75% were female, median age at drug start was 33 years (IQR 24–41) and the majority had polyarticular-course JIA (74%) (table 1). Only 92 (21%) were listed as having JIA within the BSRBR-RA data set, the remainder were listed as having an adult diagnosis. In total, 46% of patients reported at least one comorbidity, with depression (19%) and hypertension (17%) the most common. Four patients reported a remote prior malignancy.

After 1 year of starting TNFi therapy, median DAS28 improved from 6.2 (IQR 5.5–7.0) to 3.8 (IQR 2.6–5.0; p<0.0001), with 33% having a good EULAR response, and 49% a moderate response (table 2). A total of 27% of patients were classified as in DAS28 remission (DAS28<2.6). Median HAQ improved from 1.9 (IQR 1.5–2.4) to 1.5 (IQR 0.9–2.1; p<0.0001) with 66% of patients achieving a MCID in HAQ. Improvements were seen across all disease patterns, although patient numbers in some groups were too small to assess significance.

**Table 2:** Effectiveness outcomes in 443 adults with JIA starting first TNFi, by disease pattern category

	All (N=443)	Polyarticular (N=327)	Oligoarticular (N=7)	Systemic (N=12)	Enthesitis-related (N=53)	Psoriatic (N=44)
DAS28						
Baseline	6.2 (5.5–7.0) N=405	6.3 (5.6–7.0) N=322	3.9 (3.2–5.1) N=7	6.3 (5.8–7.1) N=12	5.9 (4.1–6.7) N=23	6.3 (5.5–6.9) N=41
1 year	3.8 (2.6–5.0) N=309	3.9 (2.8–5.0) N=252	3.4 (2.7–3.4) N=4	5.1 (4.2–5.8) N=7	2.2 (1.7–4.4) N=17	3.4 (2.4–4.3) N=29
Change in DAS28 at 1 year	−2.4 (−3.4 to −1.3)* N=297	−2.4 (−3.4 to −1.3)* N=248	−1.1 (−2.6 to −0.2) N=4	−1.6 (−3.4 to −1.1)* N=7	−2.3 (−3.8 to −1.7)* N=10	−2.8 (−3.5 to −1.6)* N=28
EULAR response (%) at 1 year	N=297	N=248	N=4	N=7	N=10	N=28
No response	53 (18)	46 (19)	2 (50)	1 (14)	1 (10)	3 (11)
Moderate	147 (49)	125 (50)	1 (25)	5 (71)	4 (40)	12 (43)
Good	97 (33)	77 (31)	1 (25)	1 (14)	5 (50)	13 (46)
DAS28 remission (%) at 1 year	83 (27) N=309	59 (23) N=252	1 (25) N=4	1 (14) N=7	11 (65) N=17	11 (38) N=29
HAQ						
Baseline	1.9 (1.5–3.4) N=403	2.0 (1.6–2.4) N=314	1.9 (1.0–2.3) N=6	2.1 (1.9–2.6) N=11	1.7 (0.8–2.0) N=32	1.9 (1.4–2.3) N=40
1 year	1.5 (1.9–2.1) N=260	1.6 (1.0–2.1) N=200	1.4 (0.4–1.5) N=3	2.1 (1.6–2.3) N=5	1.0 (0.1–1.4) N=24	1.6 (1.1–1.9) N=28
Change in HAQ at 1 year	−0.4 (−0.8 to 0.0)* N=242	−0.4 (−0.8 to 0.0)* N=196	−0.6 (−0.8 to −0.5)* N=3	−0.3 (−0.4 to −0.1) N=5	−0.5 (−0.8 to −0.1)* N=14	−0.5 (0.8 to −0.1)* N=24
HAQ MCID (%) at 1 year	159 (66) N=242	126 (64) N=196	3 (100) N=3	3 (60) N=5	10 (71) N=14	17 (71) N=24

Presenting median (IQR), including number with available data (N) where applicable.

DAS28, 28-joint Disease Activity Score; EULAR, European League Against Rheumatism; HAQ, Health Assessment Questionnaire; JIA, juvenile idiopathic arthritis; MCID, minimal clinical important difference; TNFi, tumour necrosis factor inhibitor.

*p<0.05.

**Figure 1:**
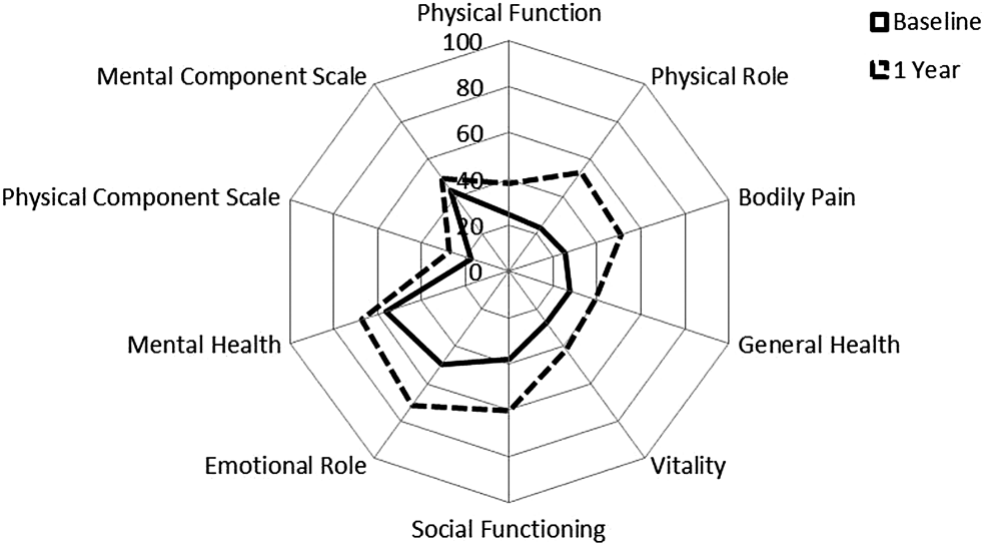
SF-36 summary and component scores in 443 adults with JIA at point of starting their first TNFi therapy and after 1 year. JIA, juvenile idiopathic arthritis; TNFi, tumour necrosis factor inhibitor; SF-36, 36-item Short Form Health Survey.

**Figure 2:**
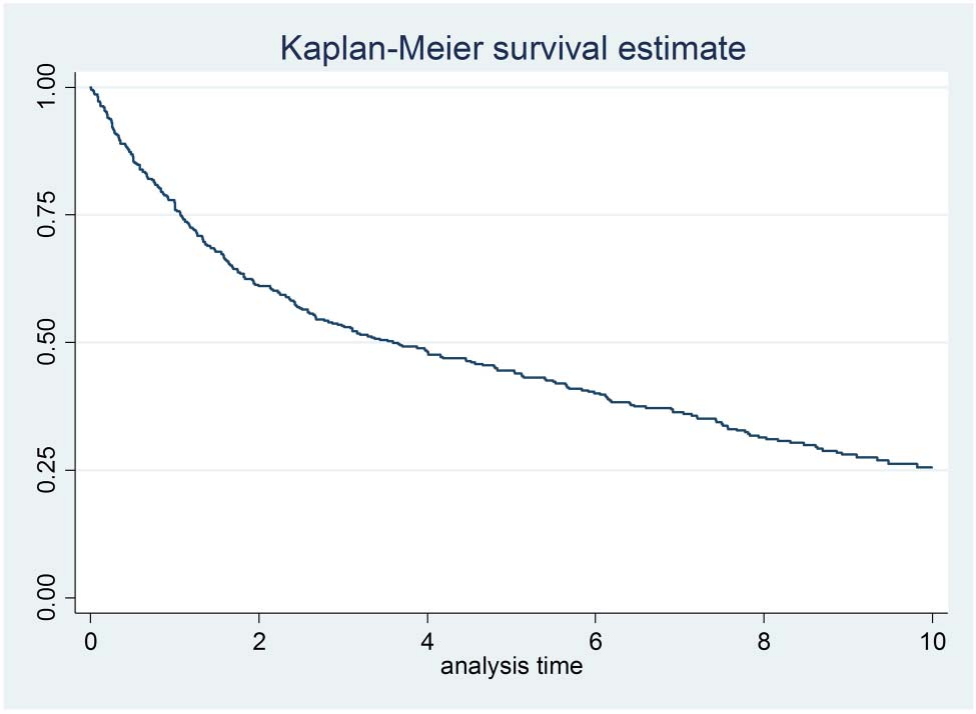
Kaplan-Meier survival estimate of time on first TNFi up to 10 years in 443 adults with JIA. JIA, juvenile idiopathic arthritis; TNFi, tumour necrosis factor inhibitor.

For the whole JIA cohort, SF-36 outcomes improved over 1 year from start of treatment; mean (SD) physical component score increased from 17.4 (9.4) to 27.4 (12.3), and mental component score increased from 43.4 (11.1) to 49.7 (10.9) (figure 1). At 3.5 years of follow-up, 50% of patients with JIA were still on first TNFi (figure 2).

Of the 443 patients with JIA starting a first TNFi, there was a mean (SD) available follow-up time of 10.0 (2.5) years per person (table 3). Total exposure time on TNFi was 2799 pyrs. There were 58 first serious infections on TNFi, incidence rate (IR) 22.3 (95% CI 17.2 to 28.8)/1000 pyrs, including 18 respiratory tract infections, 11 skin and soft tissue infections, 9 septic arthritis, 9 gastrointestinal, abdominal or reproductive infections, 6 urinary tract infections, 1 tuberculosis, 1 varicella and 3 reports of sepsis. There were four serious cardiovascular events including three arrhythmias, IR 1.4 (95% CI 0.5 to 3.8)/1000 pyrs. There were 11 reports of serious uveitis (6 on etanercept, 4 on infliximab and 1 on adalimumab), IR 4.0 (95% CI 2.2 to 7.3)/1000 pyrs. There were 16 first malignancies including 5 breast cancers, 5 carcinoma in situ of the cervix, 2 melanoma, 1 non-Hodgkin's Lymphoma, 1 non-melanoma skin cancer, 1 squamous cell carcinoma of the lung and 1 carcinoid tumour of the appendix (none of which had reported cancer prior to starting TNFi). The malignancy IR (ever exposed) was 3.7/1000 pyrs, and the SIR compared with the general population was 1.4 (95% CI 0.9 to 2.3). There were 21 deaths, mortality IR was 5.0/1000 pyrs and the SMR 2.5 (95% CI 1.7 to 3.9).

**Table 3:** Serious adverse events, malignancies and death, including crude incidence rates, SIRs and SMRs, in all adults with JIA starting first TNFi therapy

	All JIA
Total follow-up available, years	4422
Mean (SD) follow-up per person, years	10.0 (2.5)
Total exposure time on TNFi, years	2799
Mean (SD) exposure time on TNFi per person, years	6.4 (3.4)
Serious infections	58
Rate (95% CI)/1000 pyrs	22.3 (17.2 to 28.8)
Serious cardiovascular events	4
Rate (95% CI)/1000 pyrs	1.4 (0.5 to 3.8)
Uveitis	11
Rate (95% CI)/1000 pyrs	4.0 (2.2 to 7.3)
Malignancies	16
Rate (95% CI)/1000 pyrs	3.7 (2.3 to 6.0)
SIR	1.4 (0.9 to 2.3)
Deaths	21*
Rate (95% CI)/1000 pyrs	5.0 (3.2 to 7.6)
SMR	2.5 (1.7 to 3.9)

Death certificates were available in 20 of 21 deaths. The underlying causes of death were as follows: infection (4), cardiovascular disease (5: ischaemic heart disease (2), aortic stenosis (1), dilated cardiomyopathy (1), hypertensive encephalopathy (1)); end stage renal disease (1); peptic ulcer disease (1); lung cancer (1); underlying rheumatic disease (8: JIA (1); RA (5); PsA (1), AS (1)).

AS, ankylosing spondylitis; JIA, juvenile idiopathic arthritis; PsA, psoriatic arthritis; pyrs, person-years; RA, rheumatoid arthritis; SIR, standardised incidence ratio; SMR, standardised mortality ratio; TNFi, tumour necrosis factor inhibitor.

Compared with patients with RA, the 327 adults with polyarticular-course JIA were similar with respect to baseline characteristics, with the exception of longer disease duration (JIA 22 years vs RA 5 years) (table 4). At 1 year, similar improvements were seen in DAS28, HAQ and SF-36. Rates of malignancy in the JIA cohort were lower (4.3 vs 5.5/1000 pyrs) and rates of uveitis were higher (3.7 vs 0.7/1000 pyrs) compared with the RA cohort (table 5). While crude rates of serious infection were similar (23.0 and 24.6/1000 pyrs), the patients with JIA had reduced risk (adjusted HR 0.5 (95% CI 0.3 to 0.9)). Both cohorts had raised SIRs (JIA 1.6 and RA 1.9) and SMRs (JIA 2.2 and RA 3.0).

**Table 4:** Patient characteristics and disease activity measures at baseline and 1 year of 327 polyarticular JIA patients and a weighted RA cohort starting first TNFi

Median (IQR) or %	Polyarticular JIA	RA (weighted analysis)	p Value (JIA compared with RA)
Gender, female	85%	85%	1.000
Age at treatment start, years	32 (24–42)	32 (24–42)	0.928
Age at disease onset, years	13 (8–15)	25 (20–34)	0.001
Disease duration, years	22 (14–32)	5 (2–10)	0.001
Ethnicity; white	95%	93%	0.215
On methotrexate	57%	67%	0.005
On steroids	42%	42%	0.956
RF positive	46%	60%	0.001
TNFi at registration (%)			0.002
Etanercept	42	34	
Infliximab	29	26	
Adalimumab	27	32	
Certolizumab	2	7	
Total comorbidities* (%)			0.377
None	56	60	
1	30	27	
≥2	14	13	
Smoking status (%)			0.397
Current smoker	22	22	
Previous smoker	19	23	
Never smoked	59	55	
DAS28			
Baseline	6.3 (5.6–7.0)	6.3 (5.6–6.9)	0.992
1 year	3.9 (2.8–5.0)	3.8 (2.5–5.1)	0.991
Change in DAS28 at 1 year	−2.4 (−3.4 to −1.3)	−2.6 (−3.6 to −1.2)	0.589
EULAR response (%) at 1 year			0.359
No response	19	20	
Moderate	50	43	
Good	31	37	
DAS28 remission (%) at 1 year	23	26	0.539
HAQ			
Baseline	2.0 (1.6–2.4)	1.9 (1.4–2.3)	0.005
1 year	1.6 (1.0–2.1)	1.4 (0.8–2.0)	0.033
Change in HAQ at 1 year	−0.4 (−0.8 to −0.1)	−0.5 (−0.8 to −0.1)	0.523
HAQ MCID (%) at 1 year	64	66	0.775

*Comorbidities include; hypertension, angina, myocardial infarction, stroke, epilepsy, asthma, chronic bronchitis/emphysema, peptic ulcer, liver disease, renal disease, tuberculosis, demyelination, diabetes, hyperthyroidism, depression, cancer.

DAS28, 28-joint Disease Activity Score; EULAR, European League Against Rheumatism; HAQ, Health Assessment Questionnaire; JIA, Juvenile Idiopathic Arthritis; MCID, Minimally Clinical Important Difference; RA, Rheumatoid Arthritis; RF, Rheumatoid factor; TNFi, tumour necrosis factor inhibitor.

**Table 5:** Rates and relative risk of serious adverse events in adults with polyarticular JIA and RA

	Polyarticular JIA	RA (weighted analysis)
Total follow-up available (pyrs)	3228	2761
Mean (SD) follow-up per person (years)	9.9 (2.5)	8.4 (3.6)
Total exposure time to TNFi (years)	1959	1609
Mean (SD) exposure time to TNFi per person (years)	6.1 (3.5)	5.3 (3.5)
Serious infections	42	37
Rate (95% CI)/1000 pyrs	23.0 (17.0 to 31.9)	24.6 (16.9 to 37.3)
HR (95% CI)	1.0 (0.6 to 1.5)	(base)
Disease duration-adjusted HR (95% CI)	0.5 (0.3 to 0.9)	(base)
Serious cardiovascular events	3	5
Rate (95% CI)/1000 pyrs	1.5 (0.5 to 7.6)	3.0 (2.0 to 4.7)
HR (95% CI)	0.5 (0.1 to 2.2)	(base)
Disease duration-adjusted HR (95% CI)	0.3 (0.04 to 1.8)	(base)
Uveitis	7	1
Rate (95% CI)/1000 pyrs	3.7 (1.8 to 8.8)	0.7 (0.2 to 4.1)
HR (95% CI)	5.1 (0.7 to 36.2)	(base)
Disease duration-adjusted HR (95% CI)	4.7 (0.5 to 40.1)	(base)
Malignancies	13	14
Rate (95% CI)/1000 pyrs	4.3 (2.5 to 7.9)	5.5 (3.9 to 8.0)
HR (95% CI)	0.8 (0.4 to 1.6)	(base)
Disease duration-adjusted HR (95% CI)	0.4 (0.1 to 1.1)	(base)
SIR	1.6 (0.9 to 2.9)	1.9 (1.4 to 2.8)
Deaths	14	16
Rate (95% CI)/1000 pyrs	4.5 (2.7 to 8.1)	6.1 (2.7 to 16.9)
HR (95% CI)	0.7 (0.4 to 1.5)	(base)
Disease duration-adjusted HR (95% CI)	0.2 (0.1 to 0.6)	(base)
SMR	2.2 (1.3 to 4.0)	3.0 (1.3 to 8.2)

HR, hazard ratio; JIA, juvenile idiopathic arthritis; pyrs, person-years; RA, rheumatoid arthritis; SIR, standardised incidence ratio; SMR, standardised mortality ratio; TNFi, tumour necrosis factor inhibitor.

## Discussion

This analysis presents the largest observational study to date of patients with JIA starting an TNFi for the first time in adulthood. Overall, a significant improvement in disease activity was observed over the first year of therapy with a similar short-term to medium-term safety profile to that seen in an age-weighted and gender-weighted cohort of patients with RA.

There is a paucity of evidence supporting the effectiveness of TNFi started for the first time in adults with JIA. Many of these patients will have long-standing disease so may have accrued significant joint damage. In this study, despite the long disease duration, disease activity improved after 1 year of TNFi therapy; 27% patients achieved remission, as defined using the DAS28, and 33% achieved a good response. This is much higher than that reported previously in a UK-based RA cohort, with rates of 9% and 18%, respectively,^28^ although similar to the rates seen in the weighted comparison with RA included in this analysis. Younger age has been identified as a univariable predictor of remission and EULAR response in RA.^28^ Similar favourable responses were seen in HAQ and SF-36 scores, although the scores were not as high as those seen previously in an unselected cohort of adults with JIA,^5^ indicating the severe nature of disease in the patients included in this study.

One limitation in our assessment of disease activity was the necessary use of the DAS28, which has been validated in RA and PsA^29^ but not in JIA. Therefore, although the scores did improve following treatment, there will be aspects of JIA not captured, including spinal pain, enthesitis or systemic features of disease. It is also not known whether the proposed cut-off of DAS28 remission in RA equates to remission in JIA.

This study is one of the first to report SAE rates among adults with JIA receiving TNFi. The observed rate of serious infections, the most common SAE, was similar to that reported from the German JuMBO register,^11^ a cohort of adults with JIA who started biological therapies as children. The absolute risk of other SAEs was low. As we did not have access to an untreated cohort of adults with JIA receiving non-biological therapies, the rates of SAEs was compared with patients with RA using a weighted analysis to account for the different age and gender structures of the two populations. The short-term to medium-term safety profile of TNFi compared with standard therapy in RA is well characterised. The incident rate for serious infections was similar in the JIA and RA cohorts but much lower when compared with previous reports in RA.^30^ This would be expected as age is a reported independent predictor of serious infection. Investigation of risk associated with individual TNFi drugs was not possible due to small sample size.

Malignancy rates and SIRs have not previously been reported in adults with JIA. The rates are expectedly higher than those reported in children with JIA, given the higher background rate in adults. Similar to some studies in children, we did observe a possible increase in malignancy compared with the general population,^31–33^ although the numbers of any individual malignancy were too small to assess rates of specific cancers. In RA, it has been shown that the risk of lymphoma is increased, which has also been linked strongly to chronic inflammation.^34^ No study to date has looked into rates of malignancies by JIA disease pattern. The current study was able to estimate similar rates for the polyarticular disease course; however, numbers were too small to investigate in the other disease patterns. Without an untreated adult JIA cohort, it is not possible to comment on any association between TNFi and the risk of malignancy, although overall, in RA no increase in cancer has been found following TNFi therapy.^35^ Mortality rates were also increased compared with the general population, both overall and among those with polyarticular course disease. Studies of mortality in JIA, primarily in children, have been conflicting with some reporting an increase^36^ ^37^ and others reporting no difference.^38^ In part, this may relate to the challenges in studying a heterogenous disease such as JIA. The patients in this study had severe, longstanding disease requiring TNFi therapy and as such are likely to have a different outcome compared with patients with oligoarthritis who achieve remission in childhood.

Although most SAE rates were similar to or lower than that observed in RA, we did observe an increase in the occurrence of uveitis among patients with JIA, which is a recognised comorbidity of the disease.^39^ Of these 11 cases, 7 had known inflammatory eye disease at the start of therapy, although specific details or diagnosis are not captured by the BSRBR-RA. It was unfortunately not possible to tell if the reports of uveitis following therapy were new occurrences or flare of prior disease.

This is the largest study in a cohort of adults with JIA focusing on the effectiveness and safety of TNFi but does have some limitations. The majority of our patients had polyarticular course disease and therefore, our results will be less applicable to other JIA subtypes. The predominance of polyarticular course likely relates to a number of factors, including the design of the BSRBR-RA, which had a primary focus of recruiting patients with RA. Also, during the earlier years of recruitment, TNFi did not yet have a license or approval for use in the UK for conditions other than RA or children with polyarticular JIA. Indeed, many adults with JIA may have been ‘relabelled’ as having RA specifically for the purpose of gaining access to TNFi. The use of RA and other adult treatment pathways is also highlighted by the choice of infliximab and certolizumab in this population, two therapies which are not licensed in Europe for JIA. We did not have access to a comparable cohort of adults with JIA who had not received a biologic and therefore we compared outcomes with those seen in RA, where the experience with TNFi has been better characterised, including in comparison to untreated patients.^30^ ^35^ ^40^ These data cannot be used to address questions of whether TNFi increases the risk of adverse events compared with non-biological therapies in this population. Overall, it has highlighted a strong need for studies investigating long-term outcomes of adults with JIA, potentially starting from disease onset, and to encourage data collection into adulthood.

### Conclusions

The treatment pathway for adults with JIA is poorly defined, with most adults either following JIA treatment guidelines designed for children or being reclassified as adults (eg, RA, AS or PsA) to inform ongoing care. Our study has shown that for those patients with active JIA in adulthood who are naïve to biological therapy, TNFi was an effective choice of therapy in many patients with a favourable safety profile, not dissimilar to that seen in RA and provides further evidence for biological therapy use in this population. There is an ongoing need for consensus-derived care pathways to guide rheumatologists working with adults with JIA.

### Disclosures

HEF has received honoraria, educational bursaries and competitive grant awards from Pfizer. KLH reports honoraria from AbbVie, and honoraria and competitive grant awards from Pfizer. LKF salary was supported in part by a Pfizer Inflammation Competitive Research Programme Grant. All other authors have nothing to disclose.
